# Supplementary material for: Higher SARS-CoV-2 seroprevalence in workers with lower socioeconomic status in Cape Town, South Africa
Source: PLoS One. 2021 Feb 25;16(2):e0247852. doi: 10.1371/journal.pone.0247852 (PMC7906413; doi:10.1371/journal.pone.0247852)
Supplement: Attachment — Submitted filename: Response to Reviewers_PONE_11.02.2021.pdf [file pone.0247852.s001.pdf]

UNIVERSITEIT
STELLENBOSCH
UNIVERSITY

RE: PONE-D-21-00967

Higher SARS-CoV-2 seroprevalence in workers with lower socioeconomic status in Cape Town, South Africa.

Dear Godfrey Musuka, Academic Editor, PONE

Thank you for your swift response to our manuscript.

In this letter we will address each concern point-by-point. Below are the **editor, journal and reviewer's comments in bold.** Our responses are directly below each comment in normal text.

Additional Editor Comments:

Many thanks for allowing me to review this key paper which will assist the response in southern Africa in many ways. Please address the comments from the other reviewers. and also address the following comments from me:

1. **Please beef up substantially the description on the weakness of the study**

   Thank you for allowing us the opportunity to expand on this important section. The paragraph addressing the limitations of the study now reads:

   "An important limitation of this study is the moderate sample size which diminished the statistical significance of many of the findings. Furthermore, the design of the study was such that we could only sample selected participants from each sociodemographic and geographic group, who were not necessarily representative of the overall subpopulation they arose from. A further limitation of this sampling method is that it resulted in, unequal numbers from each district and occupational group, which made comparisons between groups difficult. In addition, our sample excluded those who are not part of the labour force, children, and the elderly and so can only be considered applicable to the economically active portion of the population. Our offer of serologic testing for SARS-CoV-2 before it was freely available may have introduced bias by selecting people who had a strong suspicion of having been infected. Nonetheless, we recruited a high proportion (405, 34%)

of the potential pool of 1200 volunteers and our sample included participants from a broad range of sociodemographic backgrounds, less than half of whom ever experienced symptoms of possible COVID-19 and very few of whom had contact with a known case. In hind-sight, it was felt that the participant's use of public versus private transport and the number of people in their household would have been useful sociodemographic indicators to analyse, but this data was not collected. Lastly, reported symptoms were fully reliant on participants' recollection, and *we were unable to confirm the RT-PCR results of those participants who reported having the test, which precluded further meaningful analysis of this data."*

2. **Please provide detailed recommendations for policy makers in RSA and southern Africa as a whole.**

Thank you for this opportunity.

Our 'conclusion' paragraph has now been renamed 'Conclusion and recommendations'.

The following text has been added:

"Our recommendation to policy makers in South Africa and Sub-Saharan Africa in their planning for future waves is to manage their expectations of their population, and to avoid making inferences about one subpopulation from the seroprevalence data of another. In Cape Town, strict lockdown regulations were implemented, including restrictions on businesses, educational institutions, the closure of borders to restrict traveling and a ban on all social and religious gatherings. Public health measures like the wearing of masks and an emphasis on regular sanitization of hands and surfaces were widely publicised and legally enforced. Despite this, these measures seem not to have benefitted socio-economically poorer population groups. Poor housing conditions, including crowding, and the lack of living spaces that would allow social distancing for several weeks or months, possibly contributed to a rapid spread of COVID-19 infection amongst low socio-economic strata in the population. Policy makers need to take into account that it is not reasonable to expect people whose dwellings are barely adequate as sleeping quarters and that lack any recreational space, to practice social distancing to the same degree as the better off sectors of society. Furthermore, cultural factors which drive social interactions in these areas must also be understood. This study was not designed to investigate alternative COVID-19 control measures, but the social and cultural aspects of socio-economically deprived areas will have to be considered in future epidemics when control measures are planned. It would appear, that for the

COVID-19 pandemic at least, the only likely way to protect the socioeconomically vulnerable is to ensure an early and effective vaccination strategy."

Journal requirements:

When submitting your revision, we need you to address these additional requirements.

1. **Please ensure that your manuscript meets PLOS ONE's style requirements, including those for file naming. The PLOS ONE style templates can be found at**

   https://journals.plos.org/plosone/s/file?id=wjVg/PLOSOne_formatting_sample_main_body.pdf and https://journals.plos.org/plosone/s/file?id=ba62/PLOSOne_formatting_sample_title_authors_affiliations.pdf

   Thank you, we have made appropriate modifications to the formatting of the main article, figures and references.

2. **Please provide additional details regarding participant consent. In the ethics statement in the Methods and online submission information, please ensure that you have specified:**

   - whether consent was obtained

   - whether consent was informed

   - what type of consent you obtained (for instance, written or verbal, and if verbal, how it was documented and witnessed).

   Thank you. The relevant paragraph of the Methods section now reads:

   "Each participant attended a single visit in which they underwent informed consent counselling, provided written informed consent, and data on vitals and metrics, medical history, and COVID-19 exposure and symptoms were captured directly on to an electronic database."

3. **Thank you for stating the following in the Acknowledgments Section of your manuscript:**

   "We extend our thanks to the V&A Waterfront for proposing and funding the project, and to Mr Andre Theys, his administrative team, and the V&A community advisory board for the

warm welcome and continuous support throughout its execution."

We note that you have provided funding information that is not currently declared in your Funding Statement. However, funding information should not appear in the Acknowledgments section or other areas of your manuscript. We will only publish funding information present in the Funding Statement section of the online submission form.

Please remove any funding-related text from the manuscript and let us know how you would like to update your Funding Statement. Currently, your Funding Statement reads as follows:

"The authors received no specific funding for this work."

Please include your amended statements within your cover letter; we will change the online submission form on your behalf.

Thank you for pointing this out. We welcome your advice on how to proceed with this declaration. The V&A Waterfront is not a recognised funding agency, they are a commercial entity that assisted us in this study by providing the venue for recruitment and paying for the serology assays. Our own research group undertook half the cost of the study by providing the staff and logistical support. We share the publication costs.

We have amended the acknowledgements section to: *"We extend our thanks to the V&A Waterfront for proposing the project, and to Mr Andre Theys, his administrative team…"*

With your approval, our new funding statement should read: "The project was co-funded by the V&A Waterfront Pty, Ltd and the Stellenbosch University Immunology Research Group."

Reviewers' comments:

1. Is the manuscript technically sound, and do the data support the conclusions?

Reviewer #1: Yes

Reviewer #2: Yes

2. Has the statistical analysis been performed appropriately and rigorously?

Reviewer #1: Yes

Reviewer #2: Yes

3. **Have the authors made all data underlying the findings in their manuscript fully available?**

    Reviewer #1: Yes

    Reviewer #2: Yes

4. **Is the manuscript presented in an intelligible fashion and written in standard English?**

    Reviewer #1: Yes

    Reviewer #2: Yes

5. **Review Comments to the Author**

    Reviewer #1:

    This is an interesting article and informs policy formulation in combating Covid-19. The paper looks at two aspects, firstly the SARS-CoV-2 seroprevalence in workers with lower socioeconomic status in Cape Town and then checking the specificity of the Abbott SARS-CoV-2 IgG Assay. These are very important issues as the whole world suffers from the Covid-19 pandemic.

    In the first part of the study, the authors tested 405 volunteers representing all socioeconomic strata from the workforce of a popular shopping and tourist complex in central Cape Town with the Abbott 29 SARS-CoV-2 IgG assay. They assessed the association between antibody positivity and COVID- 19 symptom status, medical history, and sociodemographic variables.

    The study found that Seropositivity was significantly associated with living in informal housing, residing in a subdistrict with low income-per household, and having a low-earning occupation. The main strength of this paper is that it addresses an interesting and timely question of whether there is an association between antibody positivity and COVID- 19 symptom status, medical history, and sociodemographic variables. It is a pertinent issue in Africa as most families have low-income jobs and live in high densely populated towns. Therefore, it was a noble idea to find out if there was a link between lower socioeconomic and SARS-CoV-2 seroprevalence.

In the second part of the study, the specificity of the Abbott SARS-CoV-2 IgG Assay. The study also noted a high specificity of the assay with regard the South African population. The sample size for the verification exercise validates the results of the assay specificity. However, it would have been good to compare the serum sample results with SARS-CoV-2 RT-PCR as it is currently the gold standard instead of assuming the absence of SARS-CoV-2 from the local population at the time of sample collection. It was also ideal to check Sensitivity of the test kit to complete the process of kit verification. This would enhance the outcome of the study as the accuracy of the test kit in the given population would have been determined.

Overall, the paper is good and concise and brings valuable information.

We thank Reviewer 1 for the positive comments.

Reviewer #2:

In general, the paper is logically presented and is easy to follow. A few spelling and grammar mistakes are present as outlined in my comments below.

The title and abstract cover the main aspects of the work. The results uncover the high seroprevalence that are not detected by the current strategies, this is novel. This provides useful insights for strategies review. The methods are clear and replicable.

All the results presented match the methods described and statistical analysis were appropriate to the research question and study design. Data presentation was clear and easy to follow. Control selection was appropriate for the study design.

There is correlation of results and conclusion. I am satisfied with the validity of the manuscript.

We thank Reviewer 2 for the positive comments.

A. Major Comment

1. **No attempt has been made to address potential bias through analytic methods, e.g., sensitivity analysis.**

   Thank you for this comment.

We performed a sensitivity analysis on the seven variables which were significantly associated with a positive serology test in the original analysis, i.e. ‘fever or chills’, ‘muscle aches’ ‘loss of smell’ and ‘loss of taste’, ‘type of dwelling’ and ‘occupation’. We intentionally misclassified a proportion of the variables in five separate scenarios ranging between 5% and 25% misclassified. We calculated the new p values using the harmonic mean p value method (HMP). In all of these five scenarios for all seven variables considered, only a single one became non-significant with intentional misclassification: ‘muscle aches’. This variable was also identified when we originally adjusted for the multiple testing effect and was noted as being non-significant after applying the Holm method.

We did not initially perform a formal sensitivity analysis because it was felt that the likelihood of such bias in this study was very low. It was an observational study with no intervention, and a single time point, therefore no opportunity for protocol deviations which might affect the outcome. The variables were all categorical, apart from age and BMI – neither of which had serious outliers which might have biased the results. The accuracy of the antibody test we used is externally validated and the cut-off point predetermined, and so could not be manipulated by us.

In our analysis we considered both pooled results, and the subgroup analysis which we have reported in the paper for the following variables: medical history (all comorbidities pooled), symptoms of COVID-19, employment type and district of residence. The results were not significantly different between the pooled analyses and the subgroup analyses.

Moreover, the analysis was conducted with the assumption that all variables were non-normally distributed.

The proportion of missing data was minimal. The only variable which had any missing data was the ‘district of residence’, where 9 (2.2%) participants were unable to be classified.

Our initial and sensitivity analysis were performed using the Fisher’s Exact Test, which was a deliberate choice as it is superior to Chi Squared when the cell numbers may be small.

We hope that the reviewers will be satisfied with the validity of our results on this basis. We have included the following sentence in the results section:

“Sensitivity analysis was performed on the seven variables which were significantly associated with a positive serology result, using five scenarios with varying proportions (5.0%, 10%, 15%, 20% and 25.0%) of variables intentionally misclassified, with the harmonic mean p value method (HMP)

used to identify a new p value for each variable. A single variable ('muscle aches') became non-significant with intentional misclassification."

B. Minor Comments.

1. **Line 99: It is important to write how serum is obtained since you cannot directly collect serum from a patient.**

   Thank you. It now reads: *"Blood collected from each participant was centrifuged, the serum divided into aliquots and frozen at -80oC."*

2. **Line 148: It is not clear if these 17 participants are out of the 180 or the whole sample of 405.**

   This line now reads: *"Only 17 of the 405 participants travelled outside of South Africa after December 2019…"*

3. **Line 151: It is not clear if these 67 are out of the total sample or 180, make it clear.**

   This line now reads: *"Sixty-seven of all 405 participants reported having…"*

4. **L-211-212-213-214. From your study there has been no significant association between designated essential workers and sero-positivity. Is it possible that in the discussion of this finding you add and highlight some studies of sero-prevalence done among essential workers such as Healthcare workers as they have been found out to be associated with risk of infection from SARS-CoV 2?**

   Thank you for the comment. We have added the following text to the discussion:
   "…and the risks of continued use of public transport during lockdown, those infected were probably infected at home rather than the workplace. However, it is likely that this is true for only the categories of essential services included in this study, as healthcare workers are likely to have a higher seroprevalence.(19,20)"

19) Varona JF, Madurga R, Peñalver F, Abarca E, Almirall C, Cruz M, et al. Seroprevalence of SARS-CoV-2 antibodies in over 6000 healthcare workers in Spain. Int J Epidemiol. 2021 Jan 12; doi.org/10.1093/ije/dyaa277

20) Goldblatt D, Johnson M, Falup-Pecurariu O, Ivaskeviciene I, Spoulou V, Tamm E, et al. Cross-Sectional Prevalence of SARS-CoV-2 antibodies in Health Care Workers in Paediatric Facilities in Eight Countries. J Hosp Infect. 2021 Jan; doi: 10.1016/j.jhin.2020.12.019

5. **This paper highlights the issues of socio-economic and demographic status in relation to SARS CoV 2 sero-positivity and as such did you by any means collect data on the races of the participants as it would be interesting to know and link to prior studies done of who is marginalised and of low socio-economic status in Western Cape and their perceived associated risk to SARS-CoV 2 infection.**

Thank you for this comment, and the opportunity to explain why we deliberately did not collect this variable.

Firstly, a racial classification system that is acceptable to both the wider scientific community, general populations and government structures is currently lacking (e.g. black vs black African vs Bantu-speaking Africans; and South African coloured vs Mixed Race vs Mixed Ancestry). For this reason self-reported ethnicity is currently preferred for scientific reports. Even self-reported ethnicity is problematic in our population, partly due to South Africa's painful history of legally enforced racial segregation, and partly due to the heterogenous multi-ethnic populations of South Africa. The current policy of Stellenbosch University and our IRB is to only report race or ethnicity if crucial to conclusions.

Secondly, too much of the debates around Covid responses have been politicized, which is why we focussed on more quantifiable, cross-cutting, less divisive classifications of socio-demographic indicators.

6. **The discussion section gave comparative analysis to UK and USA and Nigeria; please check if there are any other sero-prevalence studies in Africa, or Sub Sahara Africa that corroborates to your findings**

Thank you for the opportunity to add article which have been published subsequent to this study being written to our reference list and discussion.

The following addition has been made to the discussion:
"A recent survey of 3098 Kenyan blood donors found an overall seroprevalence of 5.6% (population-weighted seroprevalence 4.3%), which was highest in those living in the three largest urban counties of Mombasa (8.0%), Nairobi (7.3%) and Kisumu (5.5%).(28) Similar to our study, their results suggested that infection in Kenya was more widespread than the current PCR-based testing strategies would suggest."

28) Uyoga S, Adetifa IMO, Karanja HK, Nyagwange J, Tuju J, Wanjiku P, et al. Seroprevalence of anti–SARS-CoV-2 IgG antibodies in Kenyan blood donors. Science (80-). 2021 Jan 1;371(6524):79 LP – 82. doi: 10.1126/science.abe1916

C. PLOS ONE does not copyedit accepted manuscripts, so the language in submitted articles must be clear, correct, and unambiguous. Authors may consider the below small changes as applicable.

1. **Line 23: South Africa has a "higher" degree not South Africa has a "high" degree since this is comparison.**
   Changed to "Inequality if rife throughout South Africa"

2. **Line 40: Should instead be "Almost half of cases are asymptomatic" not "Half of cases are asymptomatic" because it is not exactly 50% according to your findings.**
   Changed.

3. **Line 82: Put comma (,) between "2020" and "we".**
   Changed.

4. **Line 88: should read "During the stringent lockdown" add "the".**
   Changed.

5. **Line 88: Add comma between "lockdown" and "most".**

   Changed.

6. **Line 88-89: Add comma between "work" and "either.**

   Changed.

7. **Line 98: should read "directly into" not "directly "on to".**

   Changed.

8. **Line 101: Add these commas "Abbott Laboratories, South Africa, Pty Ltd".**

   Changed.

9. **Line 103: Should be in past tense and read "An index of >1.4 was considered" not "is considered".**

   Changed.

10. **Line 105: Should read "of between 87.5% and 100%",**

    Changed.

11. **Line 105: Should read "of between 99.63% and 100%**

    Changed.

12. **Line 105: should read "Using a 99% specificity" not "Using this 99% specificity".**

    Changed.

13. **Line 107: consider the word "ascertain" not "assert".**

    Changed.

14. **Line 121-122: Should read: Out of 410 volunteers screened, 5 (4 with symptoms compatible with possible COVID-19 on the day, and 1 with an episode of syncope prior to blood draw) were excluded from the study**

    Changed.

15. **Line 134: rather "pre-existing condition" not "pre-existing diagnosis".**

    Changed.

16. **Line 143: remove "any" from "having had any".**

    Changed.

17. **Line 188: put a comma between the closing bracket and a**

    Changed.

18. **Line 204: Remove comma between "epidemic" and "demonstrates".**

    Changed.

19. **Line 218: Put a comma between "City" and "the"**

    Changed.

20. **Line 222: Put comma between "pandemic" and "the counties".**

    Changed.

21. **Line 223: Put comma between "pandemic" and "these".**

    Changed.

22. **Line 223: Put "a" between "had" and "higher".**

    Changed.

23. **Line 224: Put “a” between “UK,” and “larger”.**

    Changed.

24. **Line 227: Should be “smaller increases” not “small increases” since this is comparison.**

    Changed.

25. **Line 231: Remove comma between “wave” and “is higher”.**

    Changed.

26. **Line 232: add “a” between “with” and “high”.**

    Changed.

27. **Line 232” use “higher” and “lower” since this is a comparison.**

    Changed.

28. **Line 242: Add comma between “study” and “almost”.**

    Changed.

29. **Line 250: Add comma between “study” and “41”.**

    Changed.

30. **Line 251: Rather make it read “of which 24” instead of “of whom 24”..**

    Changed.

31. **Line 265: Should be “markers” instead of “marker” since statins is plural too.**

    Changed.

32. **Line 267: Put comma between “group” and “the”.**
    Changed.

33. **Line 269: Remove comma between “method” and “which”**
    Changed.

34. **Line 284: Put comma between “study” and “we”**
    Changed.

PLOS authors have the option to publish the peer review history of their article (what does this mean?). If published, this will include your full peer review and any attached files.

Do you want your identity to be public for this peer review? For information about this choice, including consent withdrawal, please see our Privacy Policy.

Reviewer #1: No
Reviewer #2: Yes: Dr. Tafadzwa Dzinamarira

We thank both the reviewers for their valuable input.

<u>Additional notes from the authors:</u>

We included another minor change, which we believe to be highly relevant.

In our introduction we have now referenced the recently published study from Tso et al (see below, reference 12). They assessed the rate of cross reaction between antibodies to SARS-CoV-2 spike and nucleocapsid proteins and proteins from other common coronaviruses in pre-COVID serum from Tanzania and Zambia. Their results suggest that the rate of cross reaction is high, whereas ours show the opposite.

12) Tso FY, Lidenge SJ, Peña PB, Clegg AA, Ngowi JR, Mwaiselage J, et al. High prevalence of pre-existing serological cross-reactivity against severe acute respiratory syndrome coronavirus-2 (SARS-CoV-2) in sub-Saharan Africa. Int J Infect Dis. 2020/11/08. 2021 Jan;102:577–83. doi: 10.1016/j.ijid.2020.10.104.

We thank the editor and reviewers for their time and look forward to further comments.

Regards

Dr Jane Alexandra Shaw, on behalf of the authors

Pulmonologist | Biomedical Research Institute (BMRI) Clinical Team

Division of Molecular Biology and Human Genetics

Faculty of Medicine and Health Sciences

t: +27 21 938 9639 | **a:** Fisan Building | Francie van Zijl Drive
